# Pilot study of intratumoral (IT) cryoablation (cryo) in combination with systemic checkpoint blockade in patients with metastatic melanoma (MM)

**DOI:** 10.1186/2051-1426-3-S2-P137

**Published:** 2015-11-04

**Authors:** Dae Won Kim, Cara Haymaker, Natalie McQuail, Elizabeth Sirmans, Christine Spencer, Isabella Glitza, Rodabe Amaria, Scott Woodman, Sapna Patel, Michael Davies, Cassian Yee, Wen-Jen Hwu, Chantale Bernatchez, Jennifer Wargo, Padmanee Sharma, James Allison, Patrick Hwu, Alda Tam, Adi Diab

**Affiliations:** 1 Moffitt, Tampa, FL, USA; 2 The University of Texas MD Anderson Cancer Center, Houston, TX, USA

## Background

Cryo is an effective modality for pain palliation and local control of soft tissue and bone metastases. Cryo induces necrotic cell death, and combination cryo with anti-CTLA-4 generates potent systemic anti-tumor immune responses in preclinical models and small clinical studies. However, the clinical activity of cryo plus checkpoint blockade has not been evaluated in MM. In this pilot study, we report the safety and efficacy of IT cryo and systemic ipilimumab or pembrolizumab in patients with MM.

## Methods

A single-institution study of 16 MM patients who received IT cryo during systemic therapy of checkpoint blockade. Pts with at least 1 symptomatic lesion amenable for cryo were included. Cryo was performed under ultrasound or CT-guidance. Data of symptom control, toxicity assessment and responses were collected prospectively and continued after 12 weeks of treatment administration. Exploratory immune correlates from peripheral blood and tumor samples were obtained when available.

## Results

12 out of 16 treated pts are evaluable at this time. Median age was 60 years (range: 58-82). All pts received cryo after 1 or 2 doses of either ipilimumab (N=8) or pembrolizumab (N=4). 8 patients had BRAF-V600 mutation and 3 had NRAS mutation. 10 pts had stage-IV disease (M1a: 2, M1b: 1, M1c: 7) and 2 had unresectable stage III. 4 pts were treatment naïve and 8 received 1-5 prior treatments. Objective response rates (ORR) of cryoablated (local) lesions were 75% (9/12), ORR of distant lesions were 40% (4/10) and total ORR (local+distant) were 50% (2 [with local lesions only] + 4 [distant] = 6/12) (ipilimumab-63% (5/8), pembrolizumab-25% (1/4)) by RECIST 1.1. Local disease control rates (DCR) were 83%, distant DCR were 60%, and overall DCR were 75%. With a median follow-up of 7 months, the progression-free survival rate at 6 months was 57%. At the time of the analysis, the median progression-free survival and overall survival have not been reached. Grade 3 immune related (irAE) toxicities were observed in 2 patients including colitis and hypophysitis. There were no other grade ¾ irAEs or toxicities related to cryo. Immunological analysis is ongoing.

## Conclusions

The study results suggest that the combination of cryo with checkpoint blockades is safe, well-tolerated and could potentially be an effective strategy to enhance the anti-tumor activity. These findings, warrant further evaluation in a larger prospective trial.

